# The Treatment of Syphilis

**Published:** 1897-12

**Authors:** 


### THE TREATMENT OF SYPHILIS.

Whitla (*British Medical Journal*, September 18, 1897) discussed this subject in a paper read at the recent meeting of the British Medical Association. He said that two drugs alone need be considered—mercury and the iodides. He regarded it as proved that mercury had a specific or curative effect on syphilis, and thought it best to limit his remarks to the following points: (1) How mercury and the iodides were supposed to act; (2) when should mercurial treatment be started, especially should it be given in the primary stage? (3) the various methods for its routine administration, its dosage, and the length of time necessary for mercurial treatment; (4) the treatment of tertiary symptoms and congenital syphilis. The pharmacology of mercury—the mode of absorption, especially when administered by inunction, elimination, etc.—was considered. Mercury he regarded as a vital antidote to the syphilitic poison, and so long as the virus of syphilis remained in the organism mercury, he believed, would expend its force upon it without injury to the patient. This, he thought, gave a working hypothesis as regards dosage. The question of the bactericidal power of iodides in connection with Binz's view was dealt with, and their inutility in the first and early second stages emphasized. The continuous and interrupted methods of administering mercury were treated at length although it was stated that these could not be rigidly separated. The continuous method was favored by the speaker. He prescribed small doses as early as possible. Routine treatment was deprecated. As a guide determining the effect of the mercury, the weight chart was strongly recommended. Of the various modes of administration the method of inunction was most generally useful, although this possessed many disadvantages. Under ordinary circumstances small doses of mercurious iodides, Plummer's pills, etc., were sufficient. In the tertiary stage, Dr. Whitla advised pushing the iodides until the symptoms abated. He believed that the whole secret of success in the treatment of syphilis was to get as much mercury into the system as possible without producing ill effects. He had not seen one case of harm resulting from the use of mercury in syphilis in his own practice.—*University Med. Journal.*

